# Long-term and real-life incidence of cancer therapy-related cardiovascular toxicity in patients with breast cancer: a Swedish cohort study

**DOI:** 10.3389/fonc.2023.1095251

**Published:** 2023-04-19

**Authors:** Laila Hubbert, Panagiotis Mallios, Patric Karlström, Andri Papakonstantinou, Jonas Bergh, Elham Hedayati

**Affiliations:** ^1^ Department of Cardiology and Department of Health, Medicine and Caring Sciences, Linköping University, Norrköping, Sweden; ^2^ Department of Health, Medicine and Caring Sciences, Linköping University, Linköping, Sweden; ^3^ Department of Internal Medicine, Ryhov County Hospital, Jönköping, Sweden; ^4^ Department of Oncology and Pathology, Karolinska Institute, Stockholm, Sweden; ^5^ Medical Unit: Breast, Endocrine Tumors, and Sarcoma, Theme Cancer, Karolinska University Hospital and Comprehensive Cancer Center, Stockholm, Sweden

**Keywords:** antineoplastic agents, anthracyclines, breast neoplasms, cardiovascular diseases, heart failure, hypertension, coronary artery disease, atrial fibrillation

## Abstract

**Background:**

The administration of anticancer drugs in females with comorbidity increases the risk for cancer therapy-related cardiovascular toxicity (CTR-CVT), which in turn contributes to cardiovascular disease (CVD). Furthermore, a pathophysiological connection between cancer and cardiovascular disease may exist.

**Objective:**

To assess the long-term risks and predictors of CTR-CVT, including clinical hypertension (HT), coronary artery disease (CAD), heart failure (HF), atrial fibrillation (AF), as well as all-cause mortality in women diagnosed with early breast cancer (BC) and eligible for adjuvant chemotherapy in Sweden.

**Methods:**

Data were extracted from Swedish registers and medical records on 433 women, 18-60 years of age, diagnosed 1998-2002 with lymph node-positive BC, and considered for adjuvant chemotherapy. CTR-CVT was defined as HT, CAD, HF, or AF after the diagnosis of BC. Follow-up was from the date of BC diagnosis until November 30, 2021, or death. Prevalence of CTR-CVT and all-cause mortality were calculated. Hazard ratios (HR) were determined for factors associated with CTR-CVT.

**Results:**

The median age was 50 (interquartile range (IQR) 32) years. 910 CTR-CVT events were diagnosed in 311 women with a median of 19.3 (IQR 15,3) years follow-up. The proportions of CTR-CVT events were: HT 281 (64%); CAD 198 (46%); HF 206 (47%); and AF 225 (51%). The cumulative incidence of CTR-CVT was 71.8%, and 50% of all 433 patients developed CTR-CVT within 11.7 years of BC diagnosis (standard deviation (SD) 0.57, 95% confidence interval (CI) 10.6-12.9). Age was a risk factor for CTR-CVT. Anthracycline increased the risk for HF (p=0,001; HR 2,0; 95%CI 1,4-2,8), CAD (p= 0,002; HR 1,7; 95% CI 1,2-2,4), and AF (p=0,013; HR 1,5; 95% CI 1,0-2,0). At the end of the 24-year study period, 227 of the 433 women were alive, and the total cumulative mortality was 47,6%.

**Conclusion:**

The prevalence of CTR-CVT and all-cause mortality is high after BC diagnosis and treatment, particularly in older patients and those receiving anthracyclines. These findings and the onset of CTR-CVT support cardio-oncology guidelines recommending initial risk stratification and cardiovascular monitoring during treatment, followed by long-term annual screening for cardiovascular risk factors and CTR-CVT among BC survivors.

## Introduction

Cardio-oncology comprises all forms of cardiovascular care to oncology patients before, during, and after cancer treatment. Current European Society of Cardiology guidelines on cardio-oncology recommend evaluation of cardiovascular risk factors, cardiovascular monitoring during treatment and one year, followed by long-term follow-up after breast cancer (BC) treatment (1, 2). BC is the most common form of cancer in females (3). In Sweden, the annual new case average between 2008 and 2021 was 8,600 (4). Early detection, surgery, refinement of older treatments, and the introduction of new therapies have resulted in improved outcomes (5–8). Anthracycline-based adjuvant chemotherapy such as 5-fluorouracil, doxorubicin or epirubicin, and cyclophosphamide (FAC or FEC) have been shown to reduce the relative risk of 10-year BC mortality by 20% when given in a higher cumulative dose compared to the non-anthracycline-based regimens containing including cyclophosphamide, methotrexate, and 5-fluorouracil (CMF) (6). However, at an early stage, it was discovered that anthracyclines could cause irreversible cancer therapy-related cardiovascular toxicity (CTR-CVT) (9, 10).

The individual patient data meta-analyses of randomized trials found an increase in cardiovascular (CV) mortality for anthracycline-based regimens when compared to CMF regiments (relative risk (RR) 1.50, standard error (SE) 0.38) or no chemotherapy (RR 1.61, SE=0.31). However, CV mortality did not outweigh the reduction in BC mortality (6). Other factors that increase the risk for CTR-CVT are targeted therapies with or without chemotherapy and radiotherapy (RT) that involves the heart, particularly the myocardium and coronary arteries (3, 9, 11). The administration of anticancer drugs in women with existing cardiovascular risk factors or cardiovascular disease (CVD) increases the risk for CTR-CVT. This is further increased by higher age and comorbidities such as diabetes, renal dysfunction, pulmonary disease, and endocrinopathies (1, 2). Furthermore, several common pathophysiological mechanisms linking CVD to cancer exist, including inflammation, neuro‐hormonal activation, oxidative stress, and a dysfunctional immune system (12–14). Patients with CVD and cancer contracting SARS–CoV-2 are among the highest risk groups for poor outcomes (15). The prevalence of CTR-CVT varies, and it can be induced during cancer treatment or later on (16, 17).

The aim of this study was thus to assess the long-term risk for CTR-CVT as well as all-cause mortality amongst women in Sweden diagnosed with BC and eligible for adjuvant chemotherapy between January 1998 and December 2002. The various demographic and clinical factors associated with CTR-CVT were also explored.

## Materials and methods

### Study population

Women in the Southeast Healthcare Region of Sweden (1 million inhabitants) below 60 with early lymph node-positive BC diagnosed between January 1998 and December 2002 were identified *via* the Southeast Regional Quality Registry for Breast Cancer in Sweden. The study population was chosen to capture women that fulfilled the criteria to be considered for adjuvant chemotherapy. According to regional treatment guidelines 1998-2002, only women younger than 60 with one or more lymph node metastases were eligible for adjuvant chemotherapy.

Women with primary metastatic disease, adjuvant HER2-targeted therapies, and women with known CVD and unknown cancer treatment were excluded. The cohort consisted of women with early BC treated with surgery who also received adjuvant oncological treatment. Three study groups were formed: 1) Anthracycline-containing chemotherapy (anthracycline); 2) Non-anthracycline-containing chemotherapy (other Chemo); 3) No chemotherapy (no Chemo) given. Follow-up was up to 24 years after BC surgery. By law, patients registered in national quality registries in Sweden do not need to provide written informed consent for their data to be included in healthcare research. They are notified that their data stored in registries may be removed if so wished. This study was approved by the Regional Ethics Committee (Dnr: 2012/172-31).

### Breast cancer treatment between 1998-2002

The recommended chemotherapeutic regiments that most women received were 9 cycles of anthracycline-containing chemotherapy, FEC (5-fluorouracil 600 mg/m^2^, epirubicin 60 mg/m^2^, cyclophosphamide 600mg/m^2^) or 9 cycles non-anthracycline-containing chemotherapy, CMF (cyclophosphamide 600 mg/m^2^, 5-fluorouracil 600 mg/m^2^ and methotrexate 40 mg/m^2^) both administered intravenously at 3-week intervals. Epirubicin, an anthracycline, was administered as a short infusion over 15-20 minutes.

RT to the breast was mandatory after breast-conserving surgery and sometimes to the chest wall after mastectomy. Patients with lymph node-positive disease received loco-regional RT (45-50 Gray) with the treatment goal of < 10% mean heart dose.

Adjuvant antiestrogen therapy, such as tamoxifen or aromatase inhibitors, was administered after chemotherapy and/or RT to women with an estrogen receptor-positive tumor or unknown hormone receptor status, depending on their menopausal status. Premenopausal women received 5 years of tamoxifen, a selective estrogen receptor modulator (SERM) that binds to estrogen receptors, preventing estrogen binding. Postmenopausal women received 5 years of tamoxifen or aromatase inhibitors, blocking estrogen production. In some cases, they received 2 years of tamoxifen followed by 3 years of aromatase inhibitors (switch treatment).

### Data sources

This was a retrospective registry-based cohort study where information on participants was crossmatched with various registers and medical records. Data were obtained from the Southeast Regional Quality Registry for Breast Cancer, which included data for patients from the Southeast Healthcare Region diagnosed with BC from January 1998 through December 2002. Data on the date of BC diagnosis and surgery, age at the time of BC diagnosis, laterality (right or left), tumor staging, nodal status, tumor biology (Elston Ellis grade, estrogen/progesterone status), and adjuvant treatment (chemotherapy, endocrine therapy, and RT) were retrieved from the Southeast Regional Quality Registry for Breast Cancer. Data on the type and accumulated dose of adjuvant chemotherapy, location of and total dose (Gy) of RT, and type of endocrine treatment (aromatase inhibitors or tamoxifen), baseline risk factors at the time of BC diagnosis such as obesity (BMI), diabetes mellitus, and smoking were collected from medical records. The Swedish National Board of Health and Welfare maintains the National Patient Registry (NPR) and the Swedish Cause of Death Registry (COD). The NPR collects data on inpatient (hospital) healthcare episodes and outpatient specialist care (18). The NPR and medical records were also used to retrieve data on CVD. For each patient, we recorded and categorized menopausal status, smoking habits, obesity, laterality, staging, tumor biology of the BC, and type of adjuvant oncological treatment (e.g., RT, chemotherapy, hormonal therapy, unspecified treatment, no treatment, or missing treatment data). Data on the time and cause of death were collected from the COD and medical records.

The International Classification of Diseases 10 (ICD 10) was used to retrieve data on CVD and mortality. Data from these registers and medical records were crossmatched for each patient using the unique national identification number assigned to every Swedish resident at birth or when granted permanent residency.

### Covariates

Patients were divided into 3 different age categories (≤ 40, 41-50, and 51-60 years). We also recorded and categorized menopausal status, smoking habits, obesity, laterality, staging, and tumor biology of BC, and type of oncological adjuvant treatment such as radiotherapy, chemotherapy, hormone therapy, unspecified treatment, no treatment, or missing treatment data). Left-sided BC was used as a surrogate for patients where the heart was subjected to RT.

The TNM classification system was used for tumor staging (19), but if T, N, or M data were unavailable, the tumor stage was designated missing.

### Outcome measures

The primary outcome measure used for the study was the CTR-CVT event from the time of BC surgery until the end of follow-up or death.

For this study, a CTR-CVT event was defined as hypertension (HT), coronary artery disease (CAD), heart failure (HF), or atrial fibrillation (AF) after BC diagnosis. The ICD 10 codes covered were: I 10-15 (hypertensive disease); I 20-25 (ischemic heart disease); I 50 (heart failure); and I48 (atrial fibrillation). Women with >1 CTR-CVT were included in each event category.

All-cause mortality was defined as the time (in years) between BC diagnosis and the end of follow-up (November 30, 2021) or death. Hence, for the whole cohort, data were collected over a period of at least twenty-four years.

### Statistical analysis

Patient characteristics and baseline data were summarised with descriptive statistics for the entire cohort according to the treatment group and reported as number, percentage, median and interquartile range (IQR). Cumulative incidences were reported in median and standard deviation (SD) with a 95% confidence interval (CI).

Univariable Cox proportional hazards regression was used to identify factors significantly associated with risk for CTR-CVT or all-cause mortality. A multivariate Cox analysis adjusted for, laterality, Elston Ellis Grade, estrogen receptor status, obesity, smoking, endocrine treatment, and diabetes mellitus was performed and reported for the different treatment groups and age groups regarding HT, CAD, HF, and AF. Factors with a p-value < 0.10 were initially included in a multivariable Cox regression model and reduced by backward elimination. A p-value < 0.05 indicated statistical significance and retained in the model, and hazard ratios (HR) and 95% CI were estimated. All-cause mortality was defined as the time from BC surgery to the date of death. The follow-up period was from January 1, 1998, to November 30, 2021, or death. The cumulative morbidity and mortality are illustrated by Kaplan-Meier curves and analyzed with Log Rank, Breslow, and Tarone-Ware tests. Data were analyzed and presented as descriptive and comparative statistics using IBM SPSS version 28 (Armonk, New York, USA).

## Results

**Figure 1:**
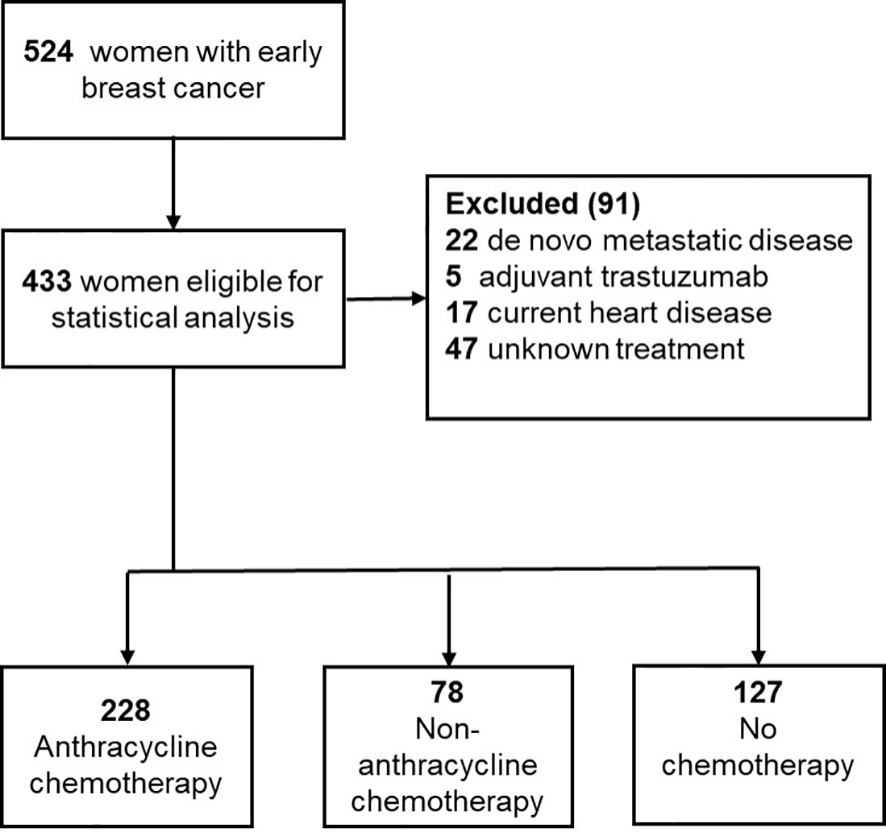
Flowchart of the data.

524 women below 60 were diagnosed with lymph node-positive early BC between January 1, 1998, and December 31, 2002, in the Southeast Healthcare Region, Sweden. Of these, 91 were excluded: 22 (4.0%) had primary metastatic disease; 5 (1.0%) received adjuvant HER2-targeted therapy within a clinical trial; 17 (3.0%) had current CVD; and 47 (9.0%) had unknown oncological treatment since the medical records (at that time, paper archives) could not be retrieved, leaving a total of 433 women that comprised the study population. Of these, 228 (53.0%) received anthracycline, 78 (18.0%) other Chemo, and 127 (29.0%) no Chemo (**Figure 1**).

For the total period of the study, the median follow-up was 19.3 (IQR 15.3) years: 21.2 years (IQR 2.4) for survivors and 5.8 years (IQR 8.4) for the deceased. The demographic and clinical baseline characteristics at BC diagnosis in the three treatment groups are listed in **Table 1**.

**Table 1:** Demographic and clinical characteristics of 433 patients^a^ with lymph node-positive early breast cancer (BC) at diagnosis in the Southeast Healthcare Region, Sweden, January 1, 1998, to December 31, 2002.

Population per study group	Anthracycline^b^ 228 (53%)	Other Chemo^c^ 78 (18%)	No Chemo^d^ 127 (29%)
**Median age,** years (IQR)	48 (11)	52 (10)	52 (10)
Age group, years			
** ≤ 40** (n=61) numbers (%) Median age, years	50 (21%) 37.5	5 (6%) 37.0	6 (5%) 37.5
** 41-50** (n=163) numbers (%) Median age, years	90 (40%) 47.0	24 (31%) 46,5	49 (39%) 47,0
** 51-60** (n=209) numbers (%) Median age, years	88 (39%) 55.2	49 (63%) 56.0	72 (57%) 57.0
Menopausal status			
Premenopausal	94 (41.6%)	19 (24.4%)	30 (23.8%)
Peri-menopausal	13 (5.8%)	4 (5.1%)	4 (3.2%)
Postmenopausal	79 (35.0%)	43 (55.1%)	63 (50.0%)
Not given	40 (17.7%)	12 (15.4%)	29 (23%)
Smoking status			
Smoker	18 (7.9%)	9 (11.5%)	3 (2.4%)
Non-smoker	90 (39.5%)	26 (33.3%)	55 (43.3%)
Ex-smoker (cessation > 6 months)	8 (3.5%)	2 (2.6%)	3 (2.4%)
Not given	112 (49.1%)	41 (52.6%)	66 (52.0%)
Obesity (BMI>30 kg/m^2^)			
Yes	16 (7.0%)	5 (6.4%)	5 (3.9%)
No	193 (84.6%)	68 (87.2%)	102 (80.3%)
Not given	19 (8.3%)	5 (6.4%)	20 (15.7%)
Laterality of BC			
Left	111 (48.7%)	40 (51.3%)	64 (50.4%)
Right	117 (51.3%)	38 (48.7%)	63 (49.6%)
Type of surgery			
Mastectomy	142 (62.3%)	50 (64.1%)	62 (48.8%)
Conservative	85 (37.3%)	28 (35.9%)	65 (51.2%)
Not given	1 (0.4%)	0	0
Type of axillary surgery			
Sentinel node	0	0	1 (0.8%)
Axilla evacuation	227 (99.6%)	77 (98.7%)	126 (99.2%)
Not given	1 (0.4%)	1 (1.3%)	0
Stage			
II	103 (45.2%)	54 (69.2%)	116 (91.3%)
III	125 (54.8%)	22 (28.2%)	10 (7.9%)
Not given	0	2 (2.6%)	1 (0.8%)
Elston Ellis grade			
Grade 1	8 (3.5%)	6 (7.7%)	27 (21.4%)
Grade 2	72 (31.6%)	25 (32.1%)	52 (41.3%)
Grade 3	120 (52.6%)	27 (34.6%)	21 (16.7%)
Not given	28 (12.3%)	20 (25.6%)	26 (20.6%)
Estrogen receptor status			
Positive	131 (57.5%)	52 (66.7%)	109 (85.8%)
Negative	89 (39.0%)	25 (32.1%)	17 (13.4%)
Uncertain	5 (2.2%)	1 (1.3%)	0
Not given	3 (1.3%)	0	1 (0.8%)
Progesterone receptor status			
Positive	130 (57.0%)	46 (59.0%)	103 (81.1%)
Negative	93 (40.8%)	31 (39.7%)	22 (17.3%)
Uncertain	2 (0.9%)	0	0
Not given	3 (1.3%)	1 (1.3%)	2 (1.6%)
Radiotherapy			
None	5 (2.2%)	1 (1.3%)	7 (5.5%)
Breast/chest wall	3 (1.3%)	1 (1.3%)	5 (3.9%)
Breast/chest wall, regional lymph nodes	139 (61.0%)	60 (76.9%)	83 (65.4%)
Breast/chest wall, regional lymph nodes, parasternal lymph nodes	76 (33.3%)	16 (20.5%)	14 (11.0%)
Axillary lymph nodes only	3 (1.3%)	0	14 (11.0%)
Not given	2 (0.9%)	0	4 (3.1%)
Hormonal treatment			
None	79 (34.6%)	22 (28.2%)	15 (11.8%)
Tamoxifen	129 (56.6%)	52 (66.7%)	101 (79.5%)
Aromatase inhibitor	11 (4.8%)	2 (2.6%)	6 (4.7%)
Switch^e^	9 (3.9%)	2 (2.6%)	2 (1.6%)
Zoladex	0	0	1 (0.8%)
Not given	0	0	2 (1.6%)

Data are presented as numbers (percentages) if not otherwise indicated.

BC, breast cancer; IQR, interquartile range; BMI, body mass index; Elston Ellis Grade, grading for breast cancer I-III.

a Patients included those who were between 18 years and 60 years old at the time of the diagnosis of eBC, had one or more lymph node metastases, fulfilled the criteria to be considered for adjuvant chemotherapy according to regional guidelines between 1998 and 2002, and did not have any cardiovascular disease prior to eBC diagnosis.

b Anthracycline-containing chemotherapy (anthracycline).

c Non-anthracycline-containing chemotherapy (other Chemo).

d No chemotherapy given (no Chemo).

e Switch: 2 years’ tamoxifen followed by 3 years’ aromatase inhibitors.

### Cancer therapy-related cardiovascular toxicity (CTR-CVT)

**Figure 2:**
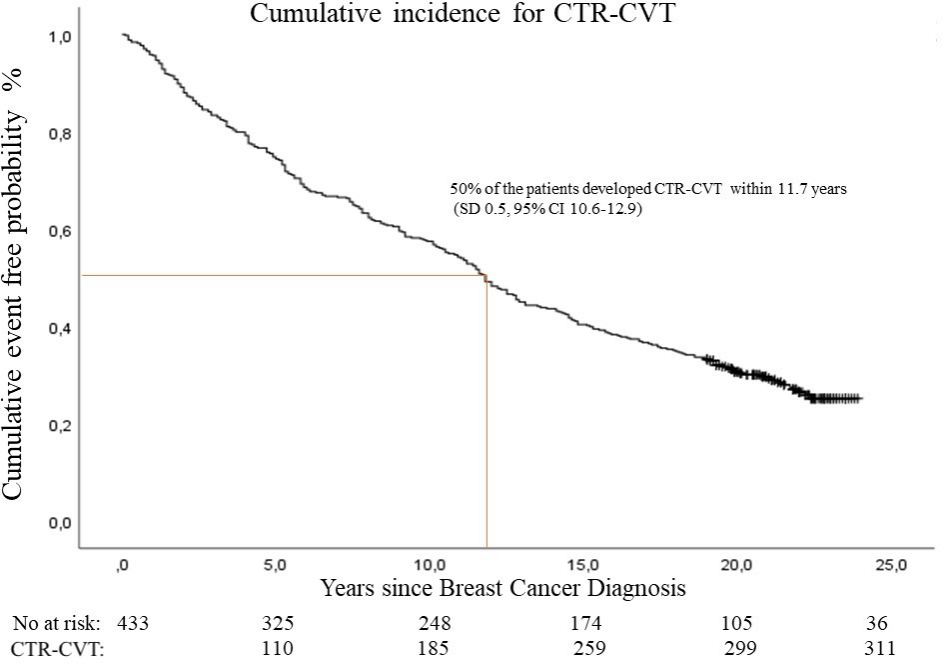
Cumulative incidence of cancer therapy-related cardiovascular toxicity (CTR-CVT) after breast cancer by years since diagnosis. 910 cancer therapy-related cardiovascular disease events in 311 patients with lymph node-positive early breast cancer at diagnosis in the Southeast Healthcare Region, Sweden, January 1, 1998, to December 31, 2002.

A total of 910 events, including HT, CAD, HF, and AF, were diagnosed in 311 women. The cumulative incidence for CTR-CVT was 71.8%, and 50.0% of the patients developed CTR-CVT within 11.7 years (SD 0.57; 95% CI 10.6-12.9) (**Figure 2**).

**Figure 3:**
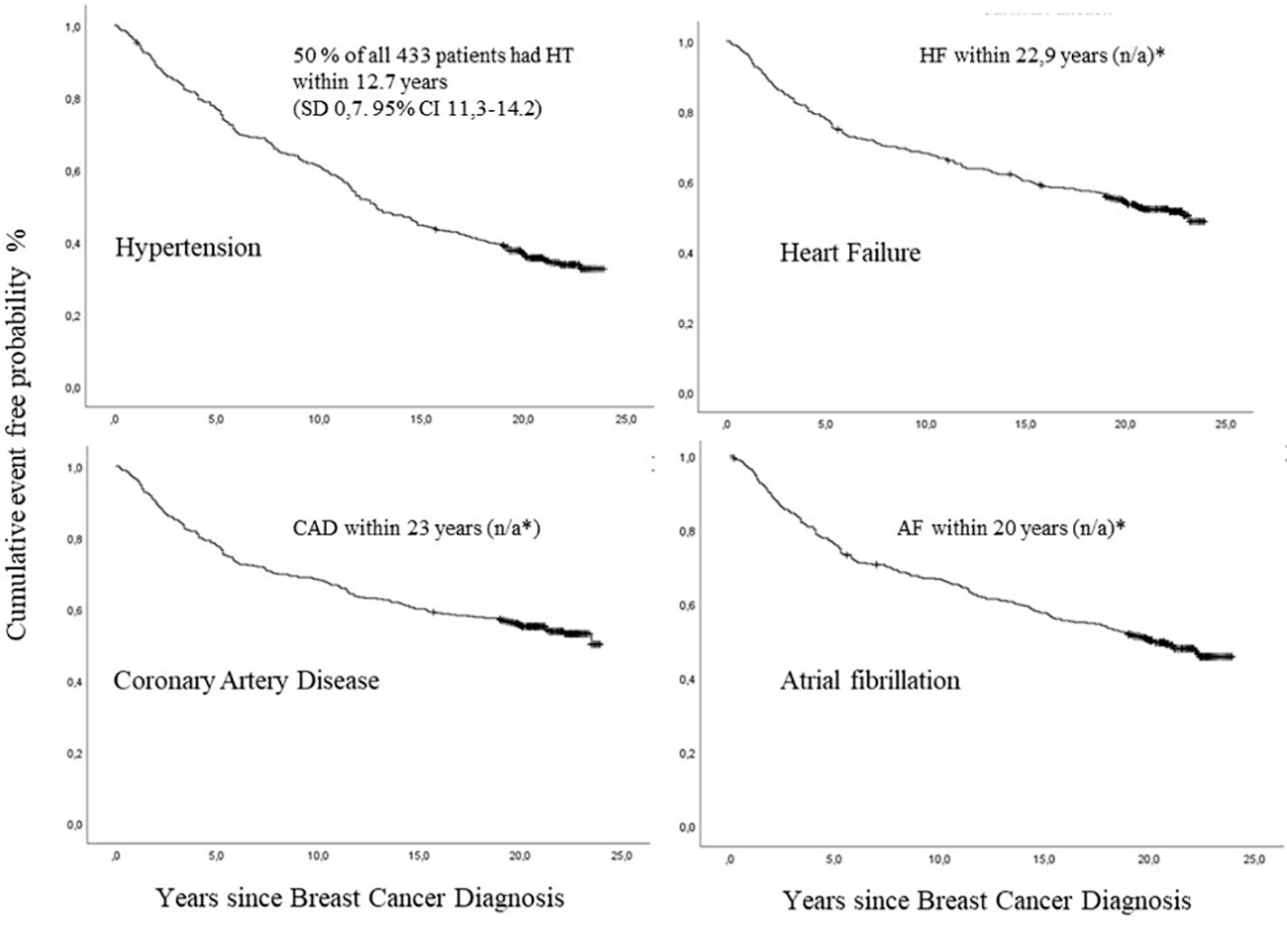
Cumulative incidence of hypertension (HT), coronary artery disease (CAD), heart failure (HF) and atrial fibrillation (AF) after breast cancer (BC) diagnosis by number of years since diagnosis, among 433 patients with lymph node-positive early breast cancer, with death as a competing risk, in the Southeast Healthcare Region, Sweden, January 1, 1998, to December 31, 2002.

The distribution of events was: anthracycline group 501 CTR-CVT events (55.1%); other Chemo group 185 events (20.3%); and the no Chemo group 224 events (24.6%) (**Table 1**). During the 24-year follow-up period, 120 (27.0%) women had no CTR-CVT, 86 women (20.0%) had one event category, 33 women (7.0%) had two categories, 18 (4.0%) had three categories, and 176 women (41.0%) suffered all four event categories (HT, CAD, HF, and AF). Cumulative incidence shows that 50.0% of all 433 patients developed HT within a median of 12.8 years (SD 0.7; 95% CI 11.3-14.2), CAD within 23 years (SD n/a), HF within 22.9 years (SD n/a), and AF within 20 years (SD n/a) (**Figure 3**).

**Figure 4:**
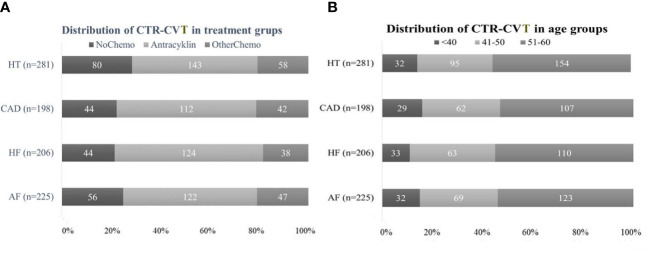
Distribution of 910 cancer therapy-related cardiovascular toxicity (CTR-CVT) events including hypertension (HT), coronary artery disease (CAD), heart failure (HF), and atrial fibrillation (AF) in 31 patients with lymph node-positive early breast cancer (BC) at diagnosis in the Southeast Healthcare Region, Sweden, January 1, 1998, to December 31, 2002. **(A)** (Left panel): Distribution of four CTR-CVT events by treatment group. Distribution of 910 CTR-CVT events in the anthracycline group 501 (55%), the other Chemo group 185 (20%), and the no Chemo 224 (25%). **(B)** (Right panel): Distribution of CTR-CVT by age group. Distribution of 910 CTR-CVT events in those ≤ 40-years-of-age 130 (14%), in those 41-50 years 289 (32%), and those 51-60 years 494 (54%).

Anthracycline treatment was associated with 51.0% of HTs, 57.0% of CADs, 60.0% of HFs, and 54.0% of AFs (**Figure 4A** **)**. The hazards ratio showed an increased risk for HF (p=0.001; HR 2.0; 95% CI 1.4-2.8), and CAD (p= 0.002; HR 1.7; 95% CI 1.2-2.4) and AF (p=0.013; HR 1.5; 95% CI 1.0-2.0), but for HT the HR was not significant (p=0.127; HR 1.2; 95% CI 0.96-1.6). The other Chemo group showed lower proportions of associated events: HT 21.0%; CAD 21.0%; HF 18.0%; and AF 21.0% (**Figure 4A**). HRs were not significant (data not shown). Distribution of 910 CTR-CVT events in those ≤ 40 years of age 130 (14%), in those 41-50 years 289 (32%), and those 51-60 years 494 (54%) (**Figure 4B**).

**Figure 5:**
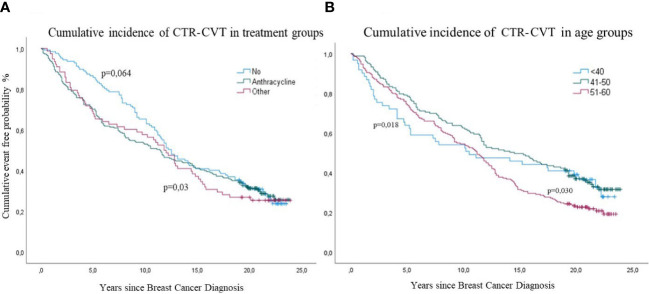
Cumulative incidence of cancer therapy-related cardiovascular toxicity (CTR-CVT) after breast cancer (BC) by years since diagnosis, with death as a competing risk, in 433 patients with lymph node-positive early breast cancer at diagnosis in the Southeast Healthcare Region, Sweden, January 1, 1998, to December 31, 2002. **(A)** (Left panel): Cumulative incidence of four CTR-CVT events by treatment group, analyzed with Log Rank (p=0,03) for all, and Breslow (p=0,06) for the no Chemo group. **(B)** (Right panel): Cumulative incidence of CTR-CVT by age group, analyzed with Log Rank (p=0,018) for all age groups and Tarone-Ware (p=0,030) for the latest years.

Cumulative incidence of four CTR-CVT events by treatment group are shown in **Figure 5A**. The highest cumulative incidence was detected in the youngest age group during the first 5-10 years (p=0.018): After that, the difference between age groups continued throughout the rest of the 24-year follow-up (p=0.030) (**Figure 5B**).

#### Time to first CTR-CVT event

**Figure 6:**
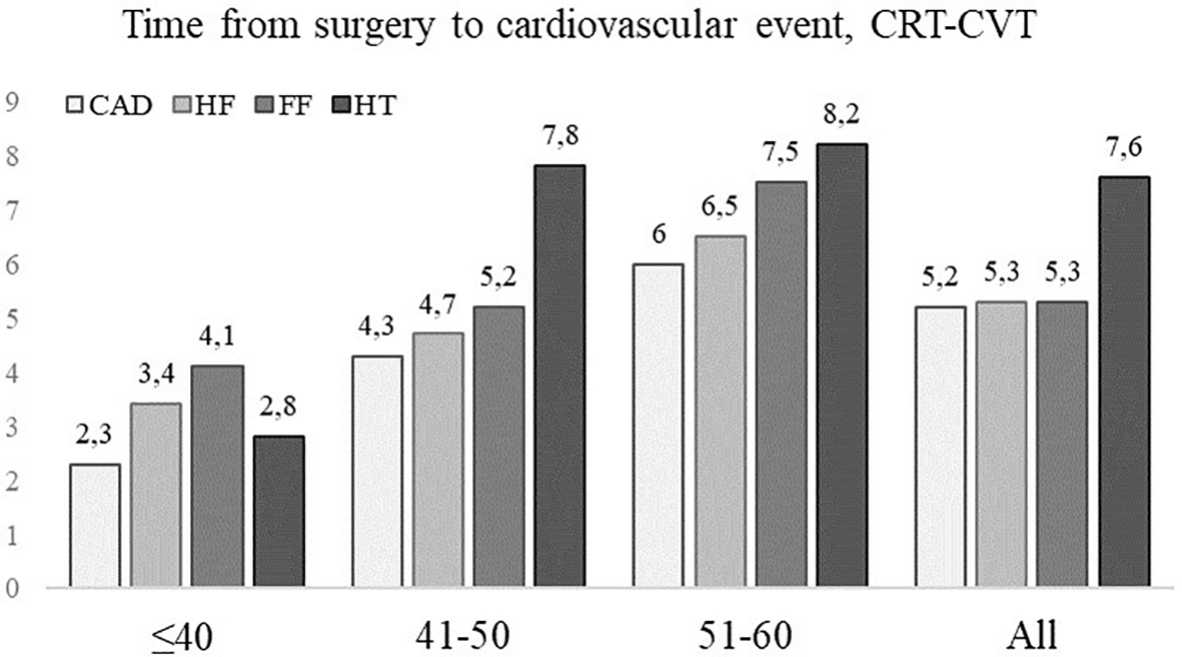
The median times to cancer therapy-related cardiovascular toxicity (CTR-CVT); hypertension (HT), coronary artery disease (CAD), heart failure (HF), and atrial fibrillation (AF) by age group after breast cancer (BC) by years since diagnosis in 433 patients with lymph node-positive early breast cancer (BC) at diagnosis in the Southeast Healthcare Region, Sweden, January 1, 1998, to December 31, 2002. Age groups: ≤ 40-years-of-age, 41-50 years, and 51-60 years.

The median time to the first CTR-CVT event after BC diagnosis was 7.6 years (SD 0.69; 95% CI 6.3-8.9). The youngest age group had its first CTR-CVT after a median of 4.1 years (SD 1.0; 95% CI 2.1-6.0), the 41-50 year group after 7.8 years (SD 1.2; 95% CI 5.4-10.1), and the oldest group (51–60) after 8.1 years (SD 0.8; 95% CI 6.2-8.9). The overall median time to the first CTR-CVT event was for HT 7.6 years (SD 0.7; 95% CI 6.1-9.0), CAD 5.2 years (SD 0.4; 95% CI 4.3-63.0), HF 5.3 years (SD 0.5; 95% CI 4.5-6.1) and for AF 5.3 years (SD 0.4; 95% CI 4.5-6.0) (**Figure 6**).

#### Hypertension

**Figure 7:**
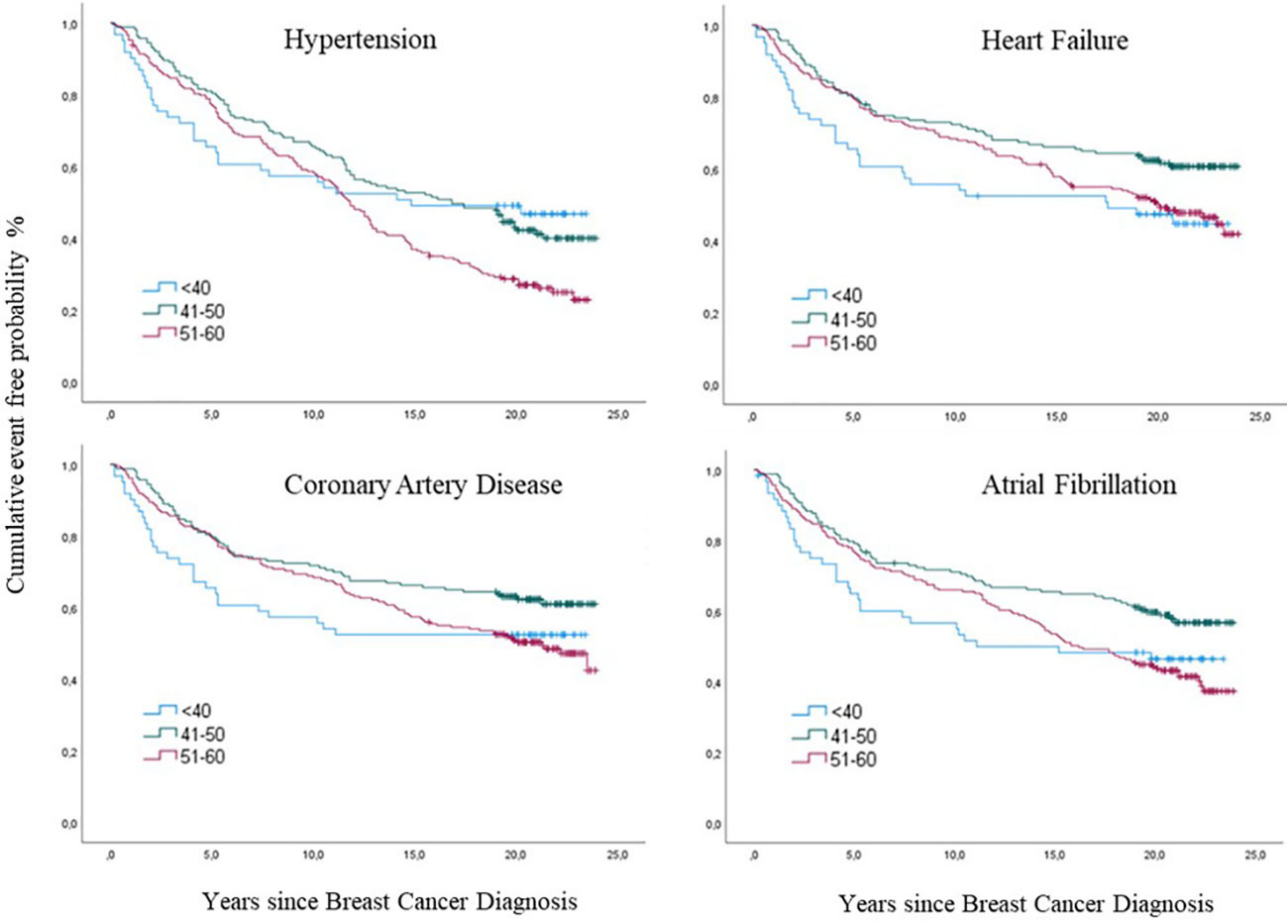
Cumulative incidences of cancer therapy-related cardiovascular toxicity (CTR-CVT); hypertension (HT), coronary artery disease (CAD), heart failure (HF) and atrial fibrillation (AF) by age group at diagnosis of breast cancer (BC) by years since diagnosis, with death as a competing Risk, in 433 patients with lymph node-positive early breast cancer at diagnosis in the Southeast Healthcare Region, Sweden, January 1, 1998, to December 31, 2002. The three age groups were ≤ 40-years-of-age, 41-50 years, and 51-60 years.

**Figure 8:**
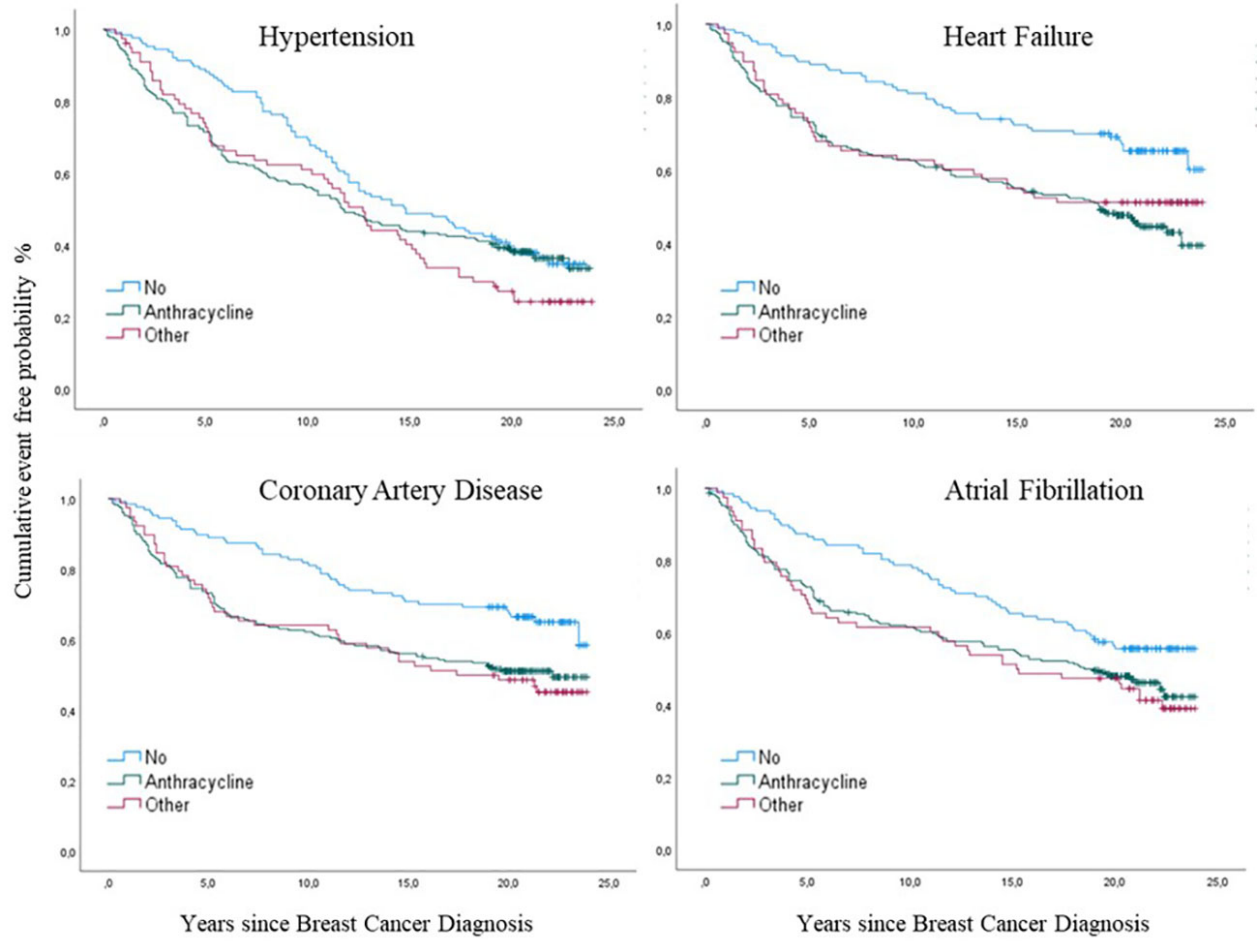
Cumulative incidences of cancer therapy-related cardiovascular toxicity (CTR-CVT); hypertension (HT), coronary artery disease (CAD), heart failure (HF) and atrial fibrillation (AF) by treatment group after breast cancer (BC) by years since diagnosis, with death as a competing risk in 433 patients with lymph node-positive early breast cancer at diagnosis in the Southeast Healthcare Region, Sweden, January 1, 1998, to December 31, 2002. Treatment groups were the anthracycline, the other Chemo, and the no Chemo groups.

Hypertension was the most prevalent event (64%), with significant differences in cumulative incidence between the three age groups (p=0.007) (**Figure 7**). In the youngest group (≤ 40), the risk for HT was higher the first eight years of observation (p=0.04) but decreased with time compared to elderly groups. The cumulative incidence of HT among the older groups did not significantly differ until after ten years. Then it became significantly higher in the oldest age group (p=0.03), where 74% had HT (**Figure 7**). The cumulative incidence of HT differed significantly between the anthracycline and the other Chemo group compared with the no Chemo group (p= 0.02) (**Figure 8**). Still, when adjusted in the multivariate analysis, there was no significantly increased risk (HR) in the anthracycline or the other Chemo group. Elston Ellis Grades II and III (histological grading of BC) were associated with significantly higher risk for HT: Grade II (p=0.047; HR 1.4; 95% CI 1.0-2.1) and Grade III (p=0.03; HR 1.7; 95% CI 1.2-2.5) compared with Grade I.

#### Coronary artery disease

CAD was seen in 198 (46%) women with no significant difference in cumulative incidence between age groups: for the youngest during the first years (p= 0.054) and for the oldest during the follow-up time (p= 0.059) and at the end of follow-up (p=0.062) (**Figure 7**). The risk for CAD was significantly higher in the other Chemo group (p=0.02. HR 1.8. 95% CI 1.1-2.7) and the anthracycline group (p=0.02. HR 1.7. 95% CI 1.2-2.4) compared to the no Chemo group (**Figure 8**). Elston Ellis Grade III was associated with a significantly higher risk for CAD (p=0.001; HR 2.3; 95% CI 1.4-3.7) compared with Grade I.

#### Heart failure

HF occurred in 206 (47%) women, where similarly increased cumulative incidences were seen in both anthracycline and other Chemo groups (p=0.001) compared to no Chemo and age (p= 0.17) (**Figure 8**). The HR for HF significantly increased with anthracycline treatment (p=0.025; HR 1.5; 95% CI 1.0-2.2). Elston Ellis Grade III was associated with a significantly higher risk for HF (p=0.008; HR 2.1; 95% CI 1.3-3.4) compared with Grade I.

#### Atrial fibrillation

AF occurred in 225 (51%) of the 443 women, with similar higher cumulative incidences in both the anthracycline and other Chemo groups (p=0.03) compared to no Chemo (**Figure 8**). Cumulative incidence was increased in the younger age group (p=0.02) (**Figure 7**). The HR for AF was higher in patients in the other Chemo group (p=0.02; HR 1.6; 95% CI 1.0-2.3) and the anthracycline group (p=0.02; HR 1.4; 95% CI 1.0-1.9) compared to no Chemo. Elston Ellis Grade III was associated with a significantly higher risk for AF (p=0.001; HR 1.9; 95% CI 1.3-2.9) compared with Grade I.

In all treatment and age groups, obesity significantly increased the risk for all CTR-CVT categories (p=0.001; HR 2.6; 95% CI 1.6-4.1), but the total number of obese patients (BMI>30 kg/m^2^) in this study was low (n=26).

None of the laterality, estrogen receptor status, smoking, endocrine treatment, and diabetes mellitus was significantly associated with a higher incidence of CTR-CVT (data not shown).

### All-cause mortality two decades after surgery

**Figure 9:**
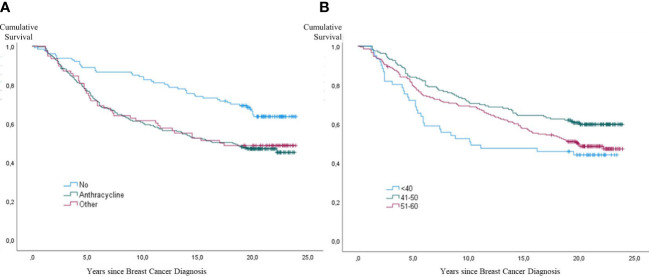
Kaplan-Meier curves showing all-cause mortality in 433 patients with lymph node-positive early breast cancer (BC) at diagnosis in the Southeast Healthcare Region, Sweden, January 1, 1998, to December 31, 2002. **(A)** (Left panel): Cumulative incidence of all-cause mortality by treatment group: Anthracycline Group, other Chemo group, and no Chemo group. **(B)** (Right panel): Cumulative incidence of all-cause mortality by age group: ≤ 40-years-of-age, 41-50 years, and 51-60 years (p < 0.001).

At the end of the 24-year study period, 227 of the 433 women were alive, and the total cumulative mortality was 47.6%. Mortality rates in the three age groups were 55.7% among those ≤ 40-years-of-age, 39.9% in patients aged 41-50 years, and 51.2% in patients 51-60 years old. The younger (≤ 40 years) and older (51-61 years) groups had significantly higher mortality rates during the observational period than the group aged 41-50 years p=0.009 and p=0.037, respectively) (**Figure 9**).

For the whole study period, the mortality risk was significantly higher in both chemotherapy groups compared to no Chemo. During the first five years of the study period, mortality was significantly higher in the anthracycline group (p=0.001; HR 3.3; 95% CI 1.6-6.8) and other Chemo group (p=0.007; HR 3.11; 95% CI 1.4-7.0) compared to no Chemo. During the almost 24-year study period, the overall mortality risk was significantly higher in both chemotherapy groups: anthracycline (p=0.001; HR 1.8; 95% CI 1.3-2.6) and other Chemo (p=0.01; HR 1.75; 95% CI 1.2-2.7) (**Figure 9**).

## Discussion

To our knowledge, this population-based Swedish cohort study is one of the largest published studies describing the prevalence of long-term chemotherapy-related cardiovascular toxicity in patients with early-stage breast cancer. It assesses whether patient- and disease-related factors, including age at the time of BC diagnosis and type of adjuvant chemotherapy, are associated with the development of CTR-CVT.

Up to 24 years after diagnosis of BC (median 19.3 years), the total cumulative incidence of all CTR-CVT events was 71.8%. Higher age at BC diagnosis, higher histological grading of BC, and adjuvant anthracycline treatment were all associated with the development of CTR-CVT after BC diagnosis. The incidence of CVD was higher compared to a population-based study comparing two Swedish cities (135 000 inhabitants each) in the same catchment area as the present study, where the mean cumulative incidence of CVD among women aged 45 to 64 years was 12%. The incidence of CAD, HF, and HT for the same populations were 4.1%, 0.95%, and 7.02%, respectively (20).

The majority of women developed all four CTR-CVT categories (HT, CAD, HF, and AF) during the study period, and HT was the most prevalent diagnosis regardless of the treatment group. The median time to CTR-CVT after BC diagnosis was 5.8 years, CAD was the earliest to develop (within 5.2 years), and HT was the latest (within 7.6 years).

The risk for CTR-CVT is twice as high in survivors of several solid cancers and lymphoma compared with the general population (21–23). The long-term real-life data in this study reveal an even higher cumulative incidence after BC, with a cumulative CTR-CVT incidence of 71.8%. The increased risk for CTR-CVT may have been due to the over-representation of postmenopausal women in the no Chemo group with already asymptomatic atherosclerotic changes or increased risk for cardiovascular disease due to shared pathophysiological mechanisms with cancer (12, 13, 16, 21). The similar incidences of CTR-CVT between age groups among women who did not receive chemotherapy compared to those receiving anthracycline might result from more prolonged survival in the no Chemo group considering the risk of CTR-CVT increases with time and age (24). However, in our study, patients with previous CVD were excluded. Further, the women in the no Chemo group were older. Thus, the risk of CVD should have been higher, and survival should have been shorter in the no Chemo group compared to other groups.

Women with aggressive BC were more likely to receive chemotherapy due to the higher risk of BC relapse and death than women with less aggressive BC who did not receive chemotherapy, which impacts the risk of CTR-CVT. However, this study included women who received relatively low doses of anthracyclines (the vast majority received epirubicin 60 mg/m^2^). This lower exposure level could explain why the risk of developing CTR-CVT was lower than expected since a dose-response relationship is believed to exist between CTR-CVT and the cumulative dose of anthracycline (25). A randomized trial between 1990 and 1998 in Sweden and Denmark, comparing the efficacy of FEC against CMF, the same chemotherapy regimens as those in our cohort, showed that the risk of CTR-CVT was similar in the two groups, as suggested by the results of this study (26).

Radiation therapy used in BC treatment increases the risk of developing

CVT, since the heart is considered radiosensitive. This risk is amplified by anthracycline therapy (21). However, radiation therapy techniques have improved since the end of the last century and the beginning of 2000, and there has been less risk for cardiac damage (27). This could explain our results of no significant increased risk for CTR-CVT among those who received left-sided RT compared to light-sided RT.

The younger age group (≤40) developed all four CTR-CVT events, and death occurred sooner, partly due to more aggressive tumors and anthracycline-containing chemotherapy as adjuvant treatment. When considering the numbers in each age group, the youngest group had similar proportions to the four CTR-CVT categories.

For all age and treatment groups, obesity was significantly associated with a higher incidence av CTR-CVT (p=0.001), but the number of patients with obesity was low.

The incidence of HT was 64% and significantly increased in the anthracycline and other Chemo groups during the first years (p=0.045). This may be due to the potential estrogen-like agonist activity and protective effect of adjuvant tamoxifen in the no Chemo group that may have delayed the development of HT (28, 29). This is consistent with our findings showing that after approximately five years, corresponding to the time of completion of tamoxifen treatment, the no Chemo group showed a similar HT incidence as the other groups. Compared with figures from the Framingham Study showing that more than 50% of 55-year-old and 66% of 65-year-old women had developed HT (30), the long-term incidence of HT in the present study could be due to age and not CTR-CVT during this long-term follow-up.

The cumulative incidence of HF of 47% was high compared to figures from the Framingham study, where cumulative incidence varied from a few percent for 50-year-old women to 10% for women aged 85 (31). Even compared to a Swedish population study showing 0.95% for women aged 45-64 years (20). Since age, treatment with an anthracycline (risk factor for HF (p=0.046)) and high Elston Ellis Grade (risk factor for HF (p=0.008)) were all found to increase the risk for HF. this comes as no surprise.

The cumulative incidence of CAD was 46% in the present study. This is higher than in 1-1.4% incidence of myocardial infarction in women 65-85 years without cancer in a report by the Swedish Public Health Department and in a regional population study showing a 4.1% incidence of CAD in women aged 45-64 years (20). Cancer has been associated with CAD development and an increased risk for recurrent myocardial infarction and major bleeding in patients with myocardial infarction (13, 32, 33). Treatment with aromatase inhibitors can induce hyperlipidemia and HT, thereby increasing the risk for CAD (34) and the impact of these risk factors on plaque progression. This is of great importance in younger patients (35).

AF had a cumulative incidence of 51%, which is high compared to the general population, with 2.7% among women aged 60-69 years and 8.1% in those 70-79 years. 5-Fluorouracil has potent CVT mediated by vascular endothelial injury and vasospasm. Our study’s high incidence of AF was most likely induced by vasospasm mediated by 5-FU (36). The cumulative incidence of CAD and AF was higher in the other Chemo group patients than those who received anthracyclines. This can be due to the other chemo group’s more prolonged survival due to better cancer prognosis than the other two groups.

Cancer has an influence on both inflammation, and the progression of CVD, in that cell proliferation is mainly driven by inflammatory molecules (37, 38). Indeed, inflammation is suggested to cause tumor initiation progression, angiogenesis, and metastasis (39). Inflammation is also paramount in the pathogenesis of atherosclerosis and CAD; inflammation and age contribute to CVD (40). Biomarkers of inflammation seem to provide prognostic information concerning cardiovascular outcomes in patients with AF (41, 42). It is possible that aggressive tumor biology, reflected in higher Elston Ellis Grading, also had an impact on inflammation and, thus, a significant impact on all CTR-CVT categories.

Of the 433 women in the study, 227 were alive after 24 years, giving a cumulative all-cause mortality of 47.6%. The younger (≤ 40 years) and the older (51-61 years) groups had significantly higher mortality during the study period compared to the 41-50 year-old group (p=0.009 and 0.037 respectively), with the highest mortality in the youngest group (p=0.001). Despite aggressive treatment, the overall mortality risk was significantly increased in both chemotherapy groups compared to the no Chemo group.

### Strengths and limitations

The study has strong internal validity because of the use of data from high-quality patient registries with low dropout rates. Furthermore, this study was on a well-defined population reflecting real-life situations, *i.e.*, the inclusion of subjects that would probably not have been included in a clinical trial, and used registries with good national coverage. The study also followed individuals over time and assessed the incidence of CTR-CVT events and their development time. These strengths suggest that our findings can be generalized to patients diagnosed with early BC living in countries with comparable breast cancer and cardiology care.

The study has several limitations, including that observational studies can only assess associations, not causal relationships. First, we did not have data regarding the socioeconomic status of the women. Thus, several factors potentially influencing the development of CTR-CVT were absent from our analyses. Future studies examining these factors would provide us with a complete understanding of whether they impact the development of CTR-CVT in patients with BC. Second, the doses and regimens used during the study period were not modern. Third, CV risk factors and CVD medication and interventions, which could protect from CTR-CVT and increase survival, were not registered, analyzed, or discussed in this study.

In summary, women under the age of 60 diagnosed with aggressive BC 1998-2002 in the Southeast Healthcare Region of Sweden had a significantly higher risk for CTR-CVT and all-cause mortality. The high incidence and the time to onset of CTR-CVT amplifies the guidelines’ recommendation of annual long-term screening for cardiovascular risk factors and CTR-CVT among BC survivors (1).

## Data availability statement

The datasets generated and/or analyzed during the current study are not publicly available because of Swedish laws and regulations, but they are available from the corresponding author upon reasonable request.

## Ethics statement

The studies involving human participants were reviewed and approved by the Declaration of Helsinki and Regional Ethics Review Board at Linköping University (Dnr: 2012/172-31). Permission was obtained to access and use the national registries and review the medical records. Written informed consent for participation was not required for this study in accordance with the national legislation and the institutional requirements. Written informed consent was not obtained from the individual(s) for the publication of any potentially identifiable images or data included in this article.

## Author contributions

EH and LH contributed to the study's conception and design. AP, LH, PM, and PK performed material preparation, data collection, and analysis. LH wrote the first draft of the manuscript, and all authors commented on subsequent versions. All authors contributed to the article and approved the submitted version.
