# Supplementary material for: Long-term and real-life incidence of cancer therapy-related cardiovascular toxicity in patients with breast cancer: a Swedish cohort study
Source: Front Oncol. 2023 Apr 19;13:1095251. doi: 10.3389/fonc.2023.1095251 (PMC10154463; doi:10.3389/fonc.2023.1095251)
Supplement: Supplementary file 2 [file Table_2.docx]

| **S Table 2.** The number of patients and the long-term incidences of hypertension, coronary artery disease, heart failure, and atrial fibrillation after BC diagnosis per age group in 433 patients^a^ with lymph node-positive early breast cancer at diagnosis in the Southeast Healthcare Region, Sweden, January 1, 1998, to December 31, 2002. | | | | |
| --- | --- | --- | --- | --- |
| ***Characteristics*** | **Patients**  n | | | |
| **CTR-CVT** | **Anthracycline^b^**  **n=228** | **Other Chemo^c^**  **n=78** | **No Chemo^d^**  **n=127** | **Total events**  **n=910** |
| Hypertension | 143 | 58 | 80 | 281 (65%) |
| Coronary Artery Disease | 112 | 42 | 44 | 198 (46%) |
| Heart Failure | 124 | 38 | 44 | 206 (48 %) |
| Atrial Fibrillation | 122 | 47 | 56 | 225 (52%) |
| Data are presented as numbers (percentages) if not otherwise indicated.  ^a^ Patients included those who were between 18 years and 60 years old at the time of the diagnosis of eBC, had one or more lymph node metastases, fulfilled the criteria to be considered for adjuvant chemotherapy according to regional guidelines between 1998 and 2002, and did not have any cardiovascular disease before eBC diagnosis.  ^b^Anthracycline-containing chemotherapy (anthracycline).  ^c^Non-anthracycline-containing chemotherapy (otherChemo).  ^d^No chemotherapy given (no-Chemo). | | | | |
